# Selective Laser Sintering of Porous Silica Enabled by Carbon Additive

**DOI:** 10.3390/ma10111313

**Published:** 2017-11-16

**Authors:** Shuai Chang, Liqun Li, Li Lu, Jerry Ying Hsi Fuh

**Affiliations:** 1 State Key Laboratory of Advanced Welding and Joining, Harbin Institute of Technology, Harbin 150001, China; msecs@nus.edu.sg (S.C.); liliqun@hit.edu.cn (L.L.); 2 Department of Mechanical Engineering and Centre for Additive Manufacturing, National University of Singapore (NUS), Singapore 117576, Singapore; mpeluli@nus.edu.sg

**Keywords:** selective laser sintering (SLS), porous ceramic, carbon additive, laser absorptivity

## Abstract

The aim of this study is to investigate the possibility of a freeform fabrication of porous ceramic parts through selective laser sintering (SLS). SLS was proposed to manufacture ceramic green parts because this additive manufacturing technique can be used to fabricate three-dimensional objects directly without a mold, and the technique has the capability of generating porous ceramics with controlled porosity. However, ceramic printing has not yet fully achieved its 3D fabrication capabilities without using polymer binder. Except for the limitations of high melting point, brittleness, and low thermal shock resistance from ceramic material properties, the key obstacle lies in the very poor absorptivity of oxide ceramics to fiber laser, which is widely installed in commercial SLS equipment. An alternative solution to overcome the poor laser absorptivity via improving material compositions is presented in this study. The positive effect of carbon additive on the absorptivity of silica powder to fiber laser is discussed. To investigate the capabilities of the SLS process, 3D porous silica structures were successfully prepared and characterized.

## 1. Introduction

Advanced ceramic materials offer great potential for high-end applications due to their unique properties such as high melting point, exceptional mechanical strength, superior wear and thermal resistance, and excellent chemical stability [1,2]. Tailored porous ceramic exhibits more special features including low thermal mass, low thermal conductivity, high surface area, low density, and high specific strength. Therefore, ceramics containing controlled porosity nowadays find many applications as end products, especially for operation environments where high temperature, extensive wear, and corrosive media are involved. The increasing number of applications for such materials include, for example, the filtration of hot corrosive gases and molten metals, high-temperature thermal insulation, catalytic carriers in various chemical processes, and membranes for separation and purification [3,4,5].

Despite their excellent properties, the wider use of porous ceramics is still limited because traditional manufacturing processes (e.g., dry pressing, slip casting, tape casting, and injection molding) are time-consuming and shaping through molding is limited, particularly for complex parts [6,7,8]. However, recent developments in additive manufacturing (AM) technologies allows for a rapid freeform fabrication of parts with complex geometries that could be very difficult or even impossible to fabricate by the conventional techniques. AM in general and selective laser sintering (SLS) in particular have recently gained popularity because of their ability to produce complex porous ceramic parts directly without a mold [9]. Manufacturing of complex polymeric or metallic parts directly has been well studied. However, there are very few works on the direct selective laser sintering (SLS) of ceramics without the use of a polymer binder. This is still a very challenging task due to the brittleness, and low thermal shock resistance properties of the material [10,11]. Besides, the key obstacle for SLS of such materials is that oxide ceramics usually show very poor absorptivity to near-infrared laser [12]. Several researchers attempted to obtain the direct SLS of ceramics through the addition of a powder coating to low melting point ceramics or by using ceramic composites with lower melting points [13]. However, the laser absorptivity of the ceramic powder coated with some transparent material such as silica was found to be even weaker [14]. Additionally, silica parts built by direct laser melting or sintering have been examined to fabricate complex tooling via sandcasting. The low purity of the silica sand used in the study resulted in a reduced melting temperature, and very poor mechanical strength. The large pores and cracks also limited its practical use [15].

Thus, this work aims to investigate the capabilities of a rapid fabrication of porous ceramic parts via the SLS technique. In general, the parts that had higher green density after SLS also exhibited higher strength. The higher the green strength, the easier complex shaped parts could be fabricated without the risk of breaking. Therefore, the direct SLS of porous ceramics remains challenging [16]. CO_2_ laser and Nd:YAG fiber laser are generally used in most SLS/M machines. In comparison to Nd:YAG fiber laser emitting at 1070 nm, CO_2_ laser with a wavelength of 10.6 μm is much more easily absorbed by most ceramic materials, although the diameter of CO_2_ laser spot is much larger than that of the fiber laser [17,18]. Therefore, the fiber laser is more suited for processing with higher accuracy. However, silica is almost non-absorbent to Nd:YAG fiber [19,20]. This study on powder absorptivity to laser aims to improve the process of selective laser sintering (SLS), and it improves our understanding of the mechanism of the interaction between laser and materials, which is crucial to finding a more uniform and suitable processing window for SLS [21]. Therefore, a solution of improving ceramic materials’ absorptivity to laser is proposed. To investigate the influence of a carbon additive on ceramic material, 3D test specimens are successfully fabricated and characterized via the SLS process.

## 2. Materials and Methods

### 2.1. Principle of SLS of Materials

**Figure 1:**
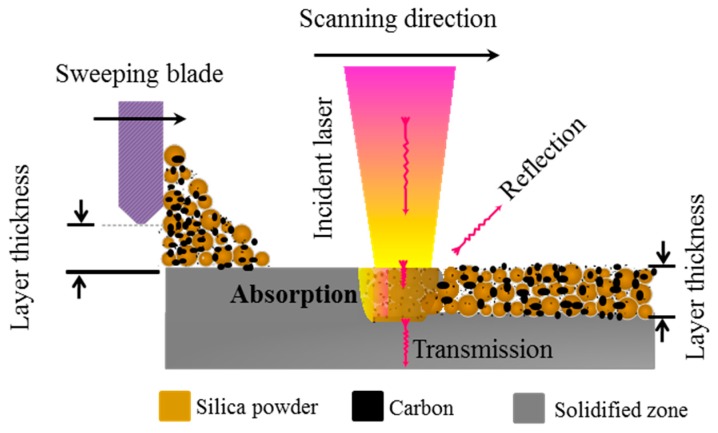
Schematic drawing of laser impinging on the powder bed in the selective laser sintering (SLS) process.

Selective laser sintering was invented by Carl R. Deckard and Joseph J. Beaman in the 1980s at the University of Texas [18]. The selective laser sintering (SLS) technique, as depicted in Figure 1, is a powder bed fusion technique in which a laser beam is used to fuse powder materials selectively according to the digital design of the built part. The CAD/CAM (computer-aided design and computer-aided manufacturing) model is sliced into thin layers with thicknesses typically less than 100 μm [22]. This technique allows for the generation of three-dimensional complex and near-net-shaped parts.

The intrinsic feature of laser processing of ceramics is that, at a certain time of interaction between the laser beam and ceramic powder bed, only a little laser energy is absorbed; some is transmitted and the rest is reflected away as depicted in Figure 1. To evaluate the efficiency of laser processing, it is necessary to know how much of the incident laser intensity is coupled to the sample. This coupling efficiency is described by “absorptance” (this is also referred to as absorption coefficient or just absorption). Other quantities often used to evaluate the laser processing are called reflectance and transmittance. The delineations of these quantities are given as follows. The absorptance, *A*, is given by:

*A* = 1 − *T*− *R* (1)

*A* = (Absorbed laser energy in the sample)/(Total incident laser power) (2)

while reflectance, *R*, is:

*R* = (Reflected laser energy from the sample)/(Total incident laser power) (3)

and transmittance, *T*, is defined by:

*T* = (Transmitted laser energy through the sample)/(Total incident laser power) (4)

In this study, reflectance (*R*) and transmittance (*T*) are measured.

### 2.2. Materials and Samples Preparation

**Figure 2:**
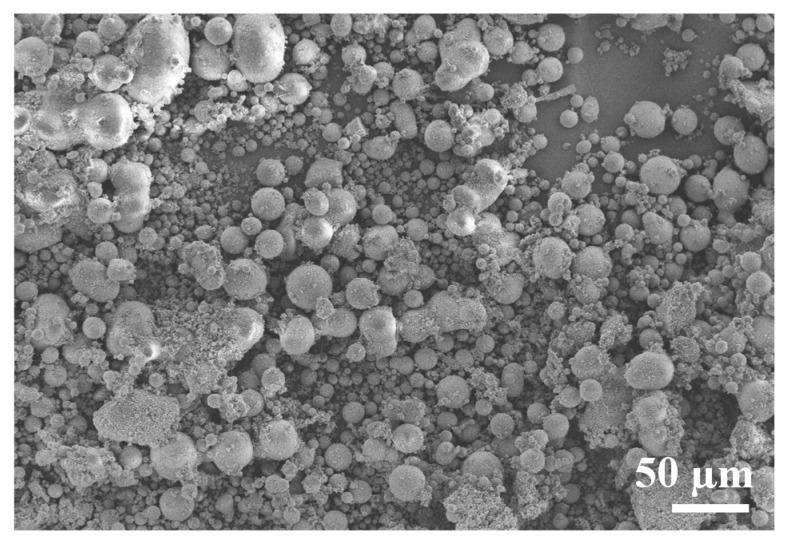
Morphology of original silica (SiO_2_) powder.

**Figure 3:**
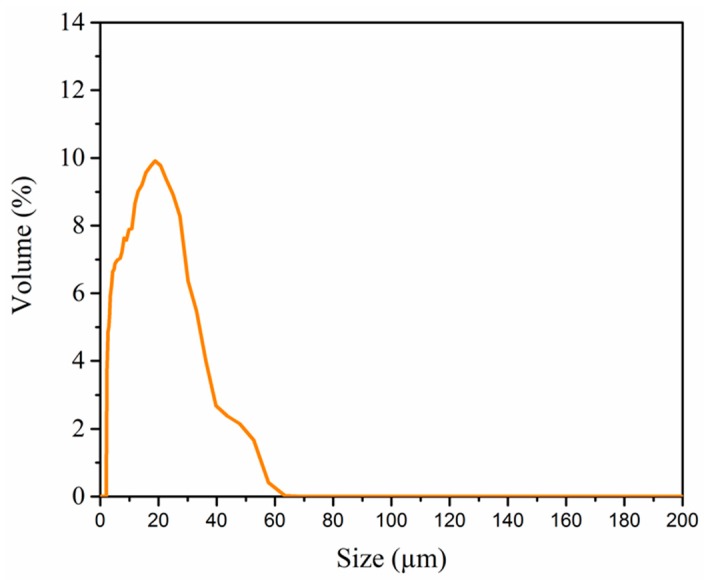
Particle size distribution of the spherical silica powder.

Silica powder (SS1206, Industrial Powder, Buffalo Grove, IL, USA) was used for this study. SEM micrograph and laser diffraction technique are used together to determine particle shape and particle size distributions. Most of the particles shown are spherical with a diameter no greater than 60 μm (Figure 2). Spherical shape is beneficial to the SLS process, since the conformation towards unity can gradually enhance both its powder packing density and flowability [23,24]. As shown in Figure 3, the particle size is distributed from 1 μm to 60 μm (measured by laser diffraction on LS 100 Q, Coulter International Corporation, West Lafayett, IN, USA). The SLS technique is more commonly utilized with typical particle size ranges of 20–150 μm for metal powder. We selected the particle size range from 1 μm to 60 μm since the melting point of ceramic is much higher than that of metals [23]. Fine active carbon powder (50–150 nm) with the mass fraction of 0.1–0.3% was doped into silica powder to increase the laser absorptivity.

Moreover, particles with a smaller size are more easily melted/reacted [23]. The composite particles were mechanically mixed with Ball Mill (from Planetary Mono Mill, FRITSCH GmbH, Idar-Oberstein, Germany) at 150 rpm for 10 h. The powder was dried in a drying oven at 80 °C for 2 h before the SLS process.

The SLS machine used in the present work is a self-developed SLS system. It is equipped with a continuous fiber laser (Pmax = 200 W.) with a wavelength of 1070 nm and a laser beam focusing diameter of ~100 μm. It is comprised of a 100 × 100 × 150 mm^3^ build envelope, and the powder layer was deposited by blade. The whole process was conducted under air atmosphere.

**Figure 4:**
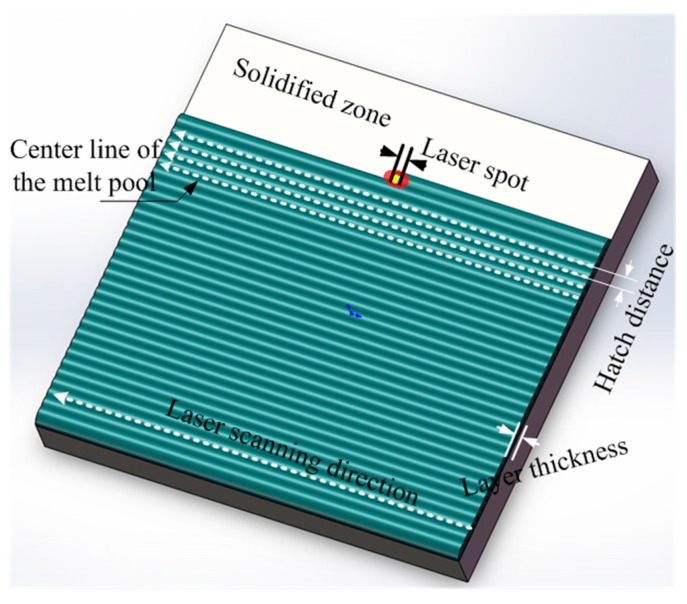
Key parameters in the SLS process.

The main parameters for the SLS processing are illustrated in Figure 4. Optimized SLS parameters shown in Table 1 were applied to fabricate green specimens with a setup dimension of 8 × 8 mm^2^ for investigations in this study.

In order to increase the bonding of the SLS parts, furnace sintering was applied in a high temperature chamber furnace (Carbolite HTF 17/5/3216P1, Chelmsford, UK). The parts were heated at 2 °C/min in air to 1200 °C for 5 h. The whole furnace sintering process is much more cost-effective and less time-consuming owing to omission of the de-binding process, because the raw material is free of polymer binder.

### 2.3. Measurements

The morphology of the specimens was observed by an optical microscope (OLYMPUS SZX10, Olympus Corporation, Tokyo, Japan) and a scanning electron microscope (SEM, JEOL JSM5510LV, Tokyo, Japan) equipped with energy dispersive X-ray spectroscopy (EDX, Oxford 7582, Oxfordshire, UK). The crystal structure was characterized by X-ray diffraction (XRD-6000 Cu-Ka radiation, Shimadzu, Tokyo, Japan). Thermogravimetric analysis and differential thermal analysis (DTA) (DTA-TG, Shimadzu DTG-60H, Shima, Tokyo, Japan) were used in the range from room temperature to 1400 °C in a flowing air atmosphere with a heating rate of 5 °C/min. The UV-vis-NIR spectra were obtained with a Cary 5000 UV-vis-NIR spectrophotometer. X-ray computed tomography (CT) scan of the printed part was carried out by using a high-resolution XCT system Phoenix Nanotom^®^ m (GE Phoenix, Lewistown, PA, USA). The density was measured by the Archimedes’ method in water. To calculate the amount of shrinkage of the SLS parts, the dimension of cubic parts was measured by digital Vernier caliper.

## 3. Results and Discussion

**Figure 5:**
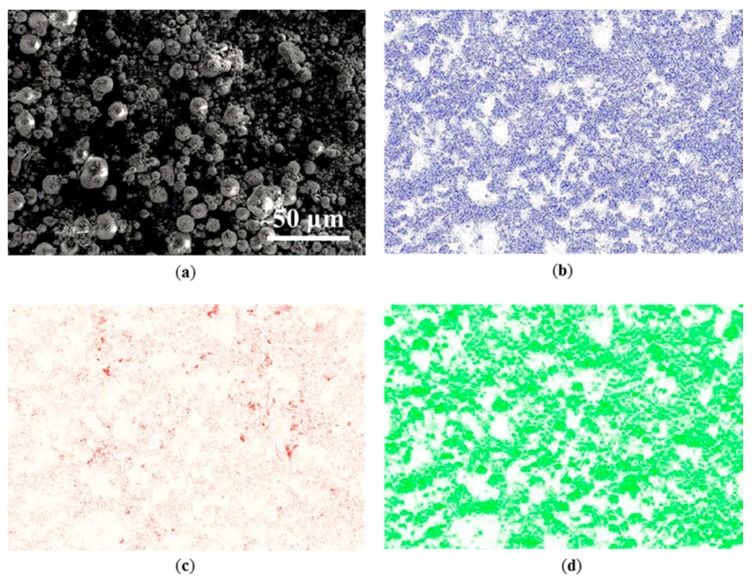
Micrographs showing characteristic distribution of silica powder with 0.2 wt % of carbon additive. (**a**) SEM micrograph of mixed powders; The corresponding EDX mapping images of (**b**) silicon; (**c**) carbon; and (**d**) oxygen elements.

SEM micrograph and EDX analyses of silica powder with carbon addition were carried out as a loose powder bed (Figure 5). The ball mill at low rotating speed achieved uniform mixing without crushing spherical silica granules (Figure 5a). The overall EDX mapping of the powder bed demonstrates that the powder contains primarily carbon (C), silicon (Si), and oxygen (O). No traces of other elements were found. This confirms that the powder was not contaminated during the ball milling process. The distribution of carbon was consistent with that of silicon and oxygen elements, which indicates that C was distributed uniformly on the micron-size particles.

**Figure 6:**
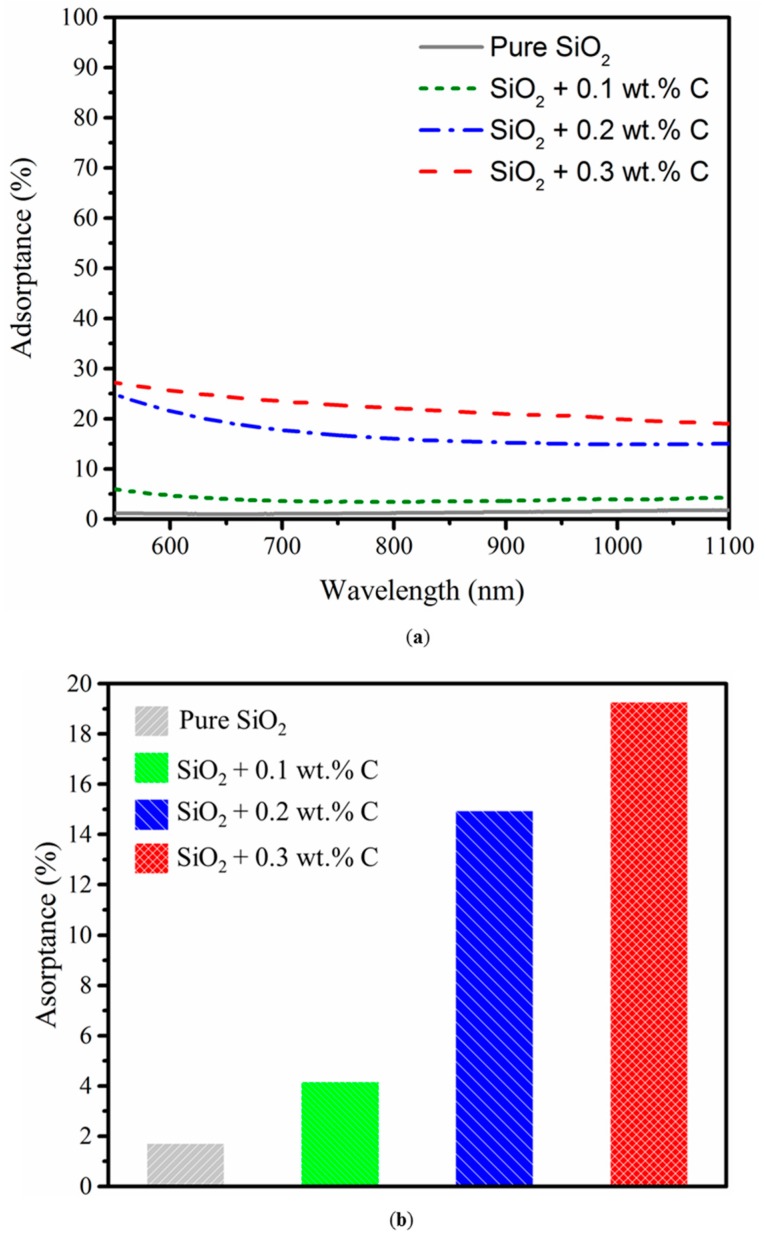
Comparison of absorptance to near-infrared light with varied amount of carbon additive. (**a**) UV-vis-NIR absorption spectra; (**b**) absorptance to UV light at 1070 nm wavelength.

As introduced previously, fine carbon was doped into the silica granules to enhance the absorptivity to laser beam. The absorptance of silica powder measured for different carbon doping quantities is presented in Figure 6. The UV-vis-NIR absorption spectra was measured on a green pellet with a thickness of 4 mm, which shows the clear improvement of absorptance by increasing amounts of carbon additive (Figure 6a). Comparison of the absorptance to fiber laser (λ = 1070 nm) in pellets containing different amount of the additive reveals significant changes (Figure 6b). The absorptance at 1070 nm wavelength of SiO_2_ + 0.1 wt % C, SiO_2_ + 0.2 wt % C, and SiO_2_ + 0.3 wt % C reached ~4%, ~16%, and ~19%, respectively. The absorbance to fiber laser of SiO_2_ + 0.2 wt % C was up to eight times higher than pure silica.

**Figure 7:**
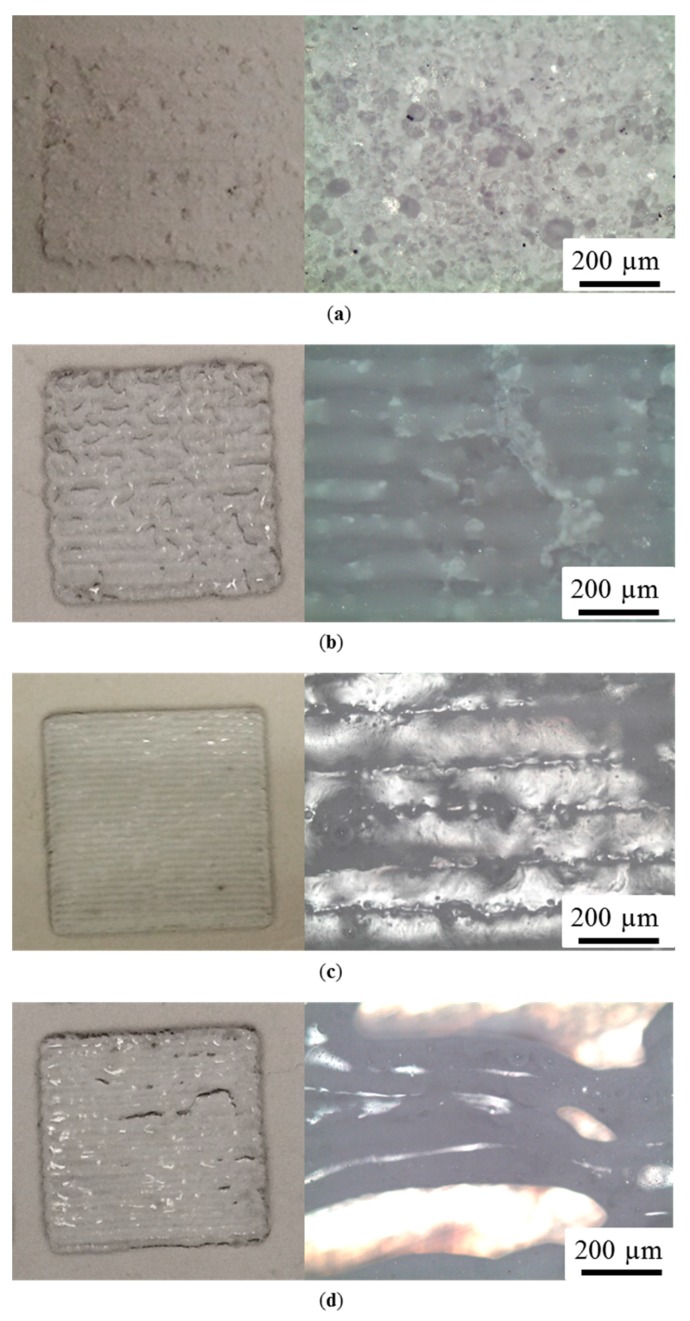
Optical graphs showing the appearance and cross-section of the SLS sample surface with varying the amount of carbon additive. (**a**) Pure silica; (**b**) silica + 0.1 wt % carbon; (**c**) silica + 0.2 wt % carbon; (**d**) silica + 0.3 wt % carbon.

The carbon nanoparticles exhibited a high tendency to absorb laser, owing to the fact that they have a much higher laser absorption, and that they have an extremely large surface area/volume ratio (i.e., because of the melting point depression phenomenon) [25]. The significant benefit from doped carbon was revealed in the comparison of the SLS of silica with different carbon additions (Figure 7). The one-layer samples shown in Figure 7 were all prepared under same process parameters, which are listed in Table 1. It is evident that the conditions of powder melting with varied carbon addition are different. The ceramic powders with carbon were at least joined to form a solid piece with the absorption of laser energy. However, there was almost no trace of melting in the pure silica powders because of poor absorptivity. It is worth mentioning that an unsuitable amount of the carbon additive would introduce macro-cracks (Figure 7d) due to excess doping, while insufficient doping may result in uncontentious joining (Figure 7b). In the range of the studied amounts of carbon additive, good qualities of melting and bonding without visible cracks were achieved by doping with 0.2 wt % of carbon (Figure 7c).

**Figure 8:**
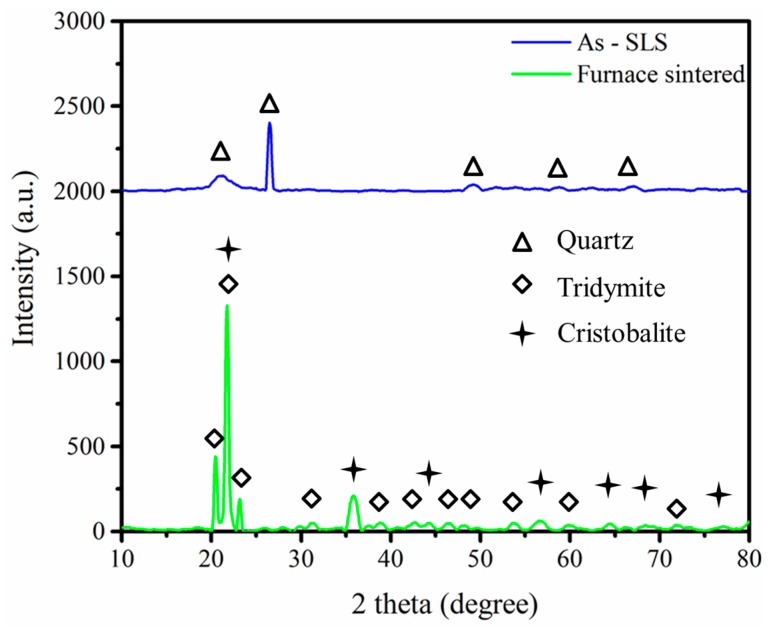
XRD pattern of the as-SLS green part and final sintered part.

**Figure 9:**
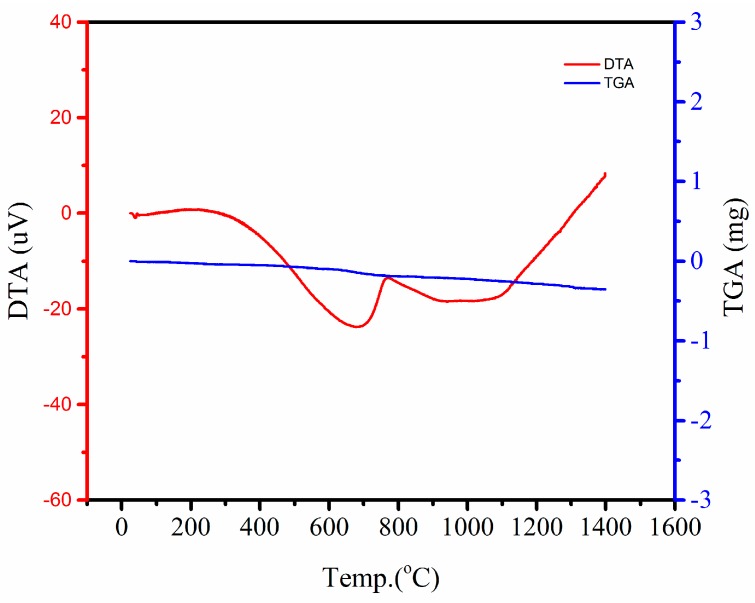
Thermogravimetric analysis and differential thermal analysis (DTA/TGA) curve of the silica powder with 0.2 wt % of carbon additive.

Along with doping a small amount of carbon, the laser absorptivity at 1070 nm was obviously enhanced. On the other hand, it was proven that no reaction with active carbon occurred, as shown in the X-ray diffraction (XRD) results (Figure 8). The X-ray diffraction pattern revealed that the structures are well crystallized into the quartz phase after laser sintering. The transformation of quartz into cristobalite and tridymide after furnace sintering was also analyzed. There is no carbon phase or any composite phase detected in both green and final ceramic parts as a consequence of carbon addition. Hence, there is no risk of inducing undesirable contamination or a new phase, which may have negative effects on the final properties. The carbon may be consumed by the reaction with the oxygen in the air, which will contribute some heat energy to the SLS process. This thermal behavior of the carbon additive is shown by the DTA/TGA curve (Figure 9).

**Figure 10:**
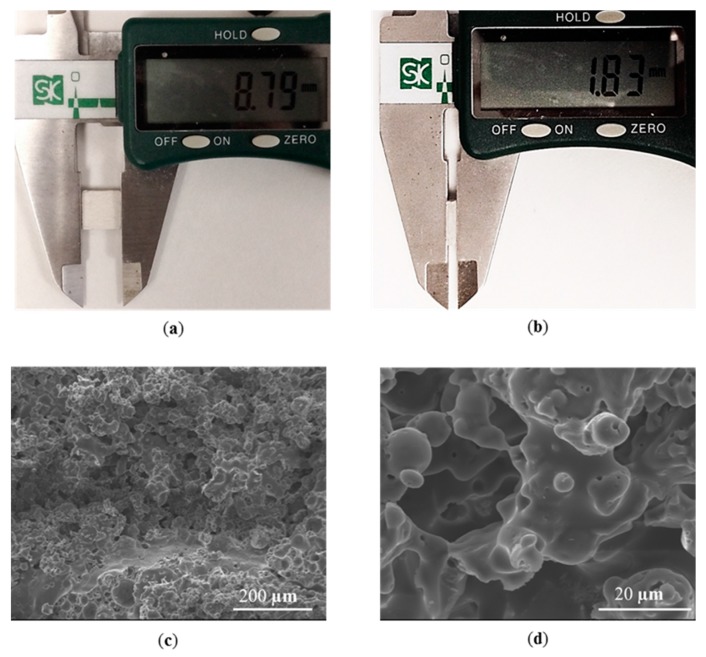
Silica part produced by the SLS + sintering. (**a**) Top view of the silica cubic; (**b**) side view of the silica cubic. SEM micrographs of fracture surface of final sintered sample; (**c**) lowmagnification; (**d**) high magnification.

**Figure 11:**
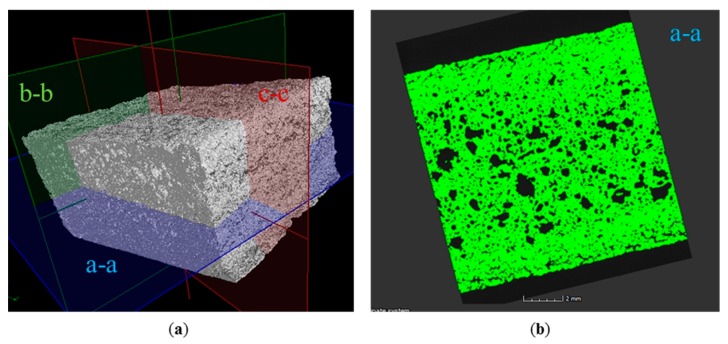

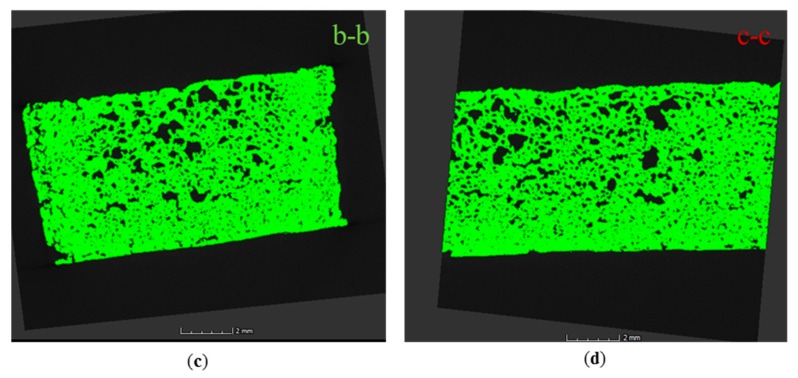
Image renderings from micro- computed tomography (CT) scans of final porous silica specimen produced by SLS. (**a**) 3D image; (**b**) cross-section parallel to the building direction; (**c**) cross-section vertical to the laser scanning direction; (**d**) cross-section parallel to the laser scanning direction.

Good melting and bonding is the prerequisite for creating porous structures. One efficient way of generating the porous part is to enlarge the hatch distance (*H*). In this case, the particles would bond together but a void could be left inside. Therefore, the porosity can be tailored by varying *H*. The fabrication of a 3D porous silica part was conducted with optimized parameters and lager hatch distance (300 μm) according to Table 1. A porous silica cubic sample with 20 layers (Figure 10) was successfully fabricated via SLS followed by furnace sintering, and the relative density was only 46.8%. Firstly, the silica sample was evaluated by visual inspection since it is quick, simple, and cheap to perform to most specimens [26], and the sample showed a porous-like and crack-free surface. Defects such as cracks were not observed in the cross-section of the sample (Figure 10c). Continuous pores with random distribution were formed between the particles, which bonded via a necking mechanism (Figure 10d). The successful fabrication of silica with tailored porosity was also demonstrated in the 3D reconstruction from the micro-CT scan (Figure 11). The linear and volume shrinkage: ΔL/L, ΔW/W, ΔH/H, and ΔV/V, were measured by digital Vernier caliper, and the results were 1.45%, 1.57%, 2.7%, and 6.75% in length, width, height, and volume, respectively. Excellent bonding and low shrinkage of the porous silica structure were obtained owing to the direct SLS with carbon additive and without the use of a polymer binder in laser sintering.

## 4. Conclusions

In this paper, the capabilities of manufacturing porous silica by means of selective laser sintering (SLS) without using a polymer binder have been demonstrated. The significant improvement of laser absorptivity by doping carbon on the silica material has been highlighted. Furthermore, there was no trace of undesirable contamination or new phase, which may result in negative effects on the final properties. The well-bonded 3D porous silica structure with low shrinkage was successfully fabricated without visible defects. Moreover, this approach is also applicable to the fabrication of other porous ceramic parts through laser sintering. The material processing technique, although time-consuming, makes the direct SLS of porous silica structures possible.

## Figures and Tables

**Table 1:** Optimized laser sintering parameters.

Laser Power (W)	Hatch Space (µm)	Scan Speed (mm/s)	Spot Size (µm)	Layer Thickness (µm)
60	150	50	120	90
